# Supplementary material for: Post-COVID conditions following COVID-19 vaccination: a retrospective matched cohort study of patients with SARS-CoV-2 infection
Source: Nat Commun. 2024 May 22;15:4101. doi: 10.1038/s41467-024-48022-9 (PMC11111703; doi:10.1038/s41467-024-48022-9)
Supplement: Supplementary file 1 — Supplementary Information [file 41467_2024_48022_MOESM1_ESM.pdf]

Supplementary Information

Post-COVID conditions following COVID-19 vaccination: A retrospective matched cohort study of patients with SARS-CoV-2 infection

Supplementary Figure 1**.** Study population flow chart for matched analysis

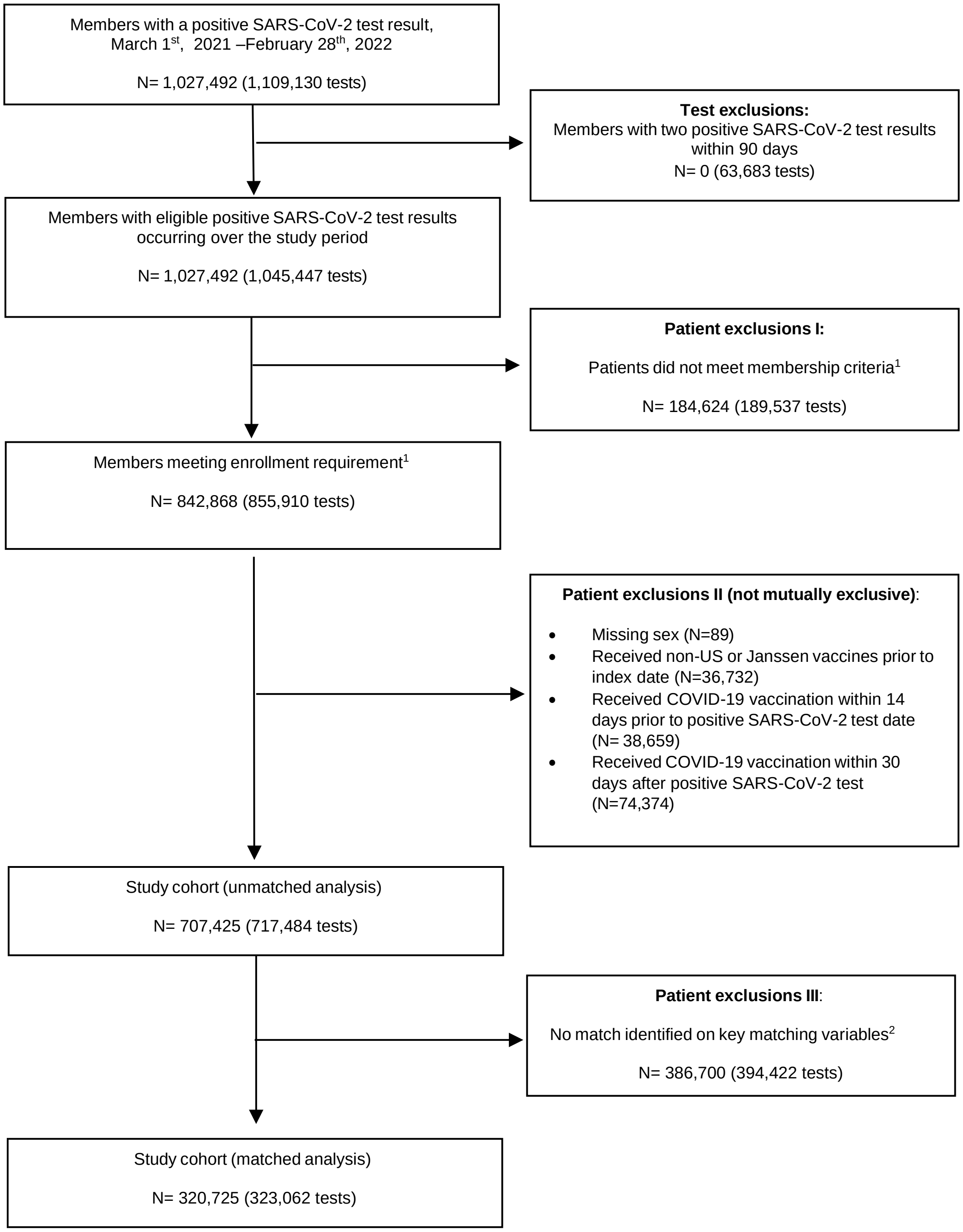

[1] Continuous healthcare membership 1 year prior and for 30 days following date of positive SARS-CoV-2 test, allowing a 31-day enrollment gap.

[2] Matching criteria included Vaccine Safety Datalink site, date of SARS-CoV-2 positive test (±7 days), age (exact year), sex, and severity of infection (hospital admission with COVID-19 diagnosis within 7 days of SARS-CoV-2 positive test)

Supplementary Figure 2. Distribution of SARS-CoV-2 tests over time, in matched (A) and unmatched (B) study cohorts

A. Matched cohort

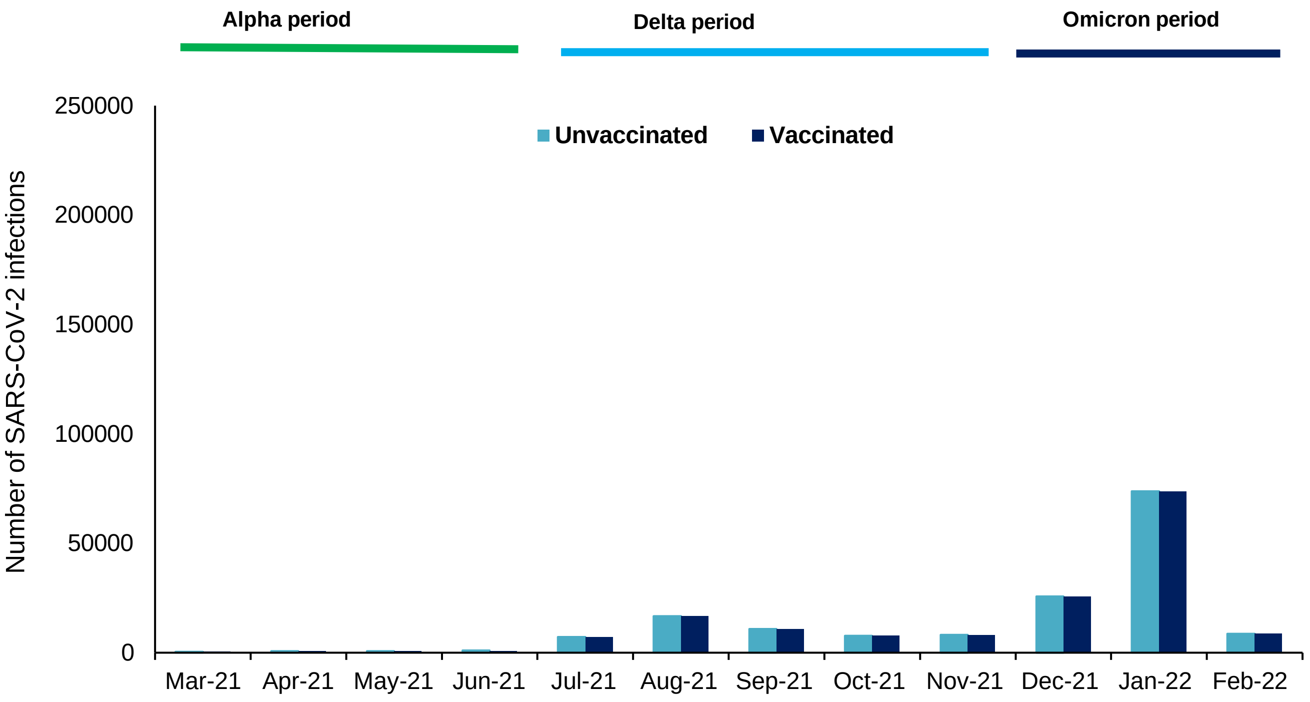

B. Unmatched cohort

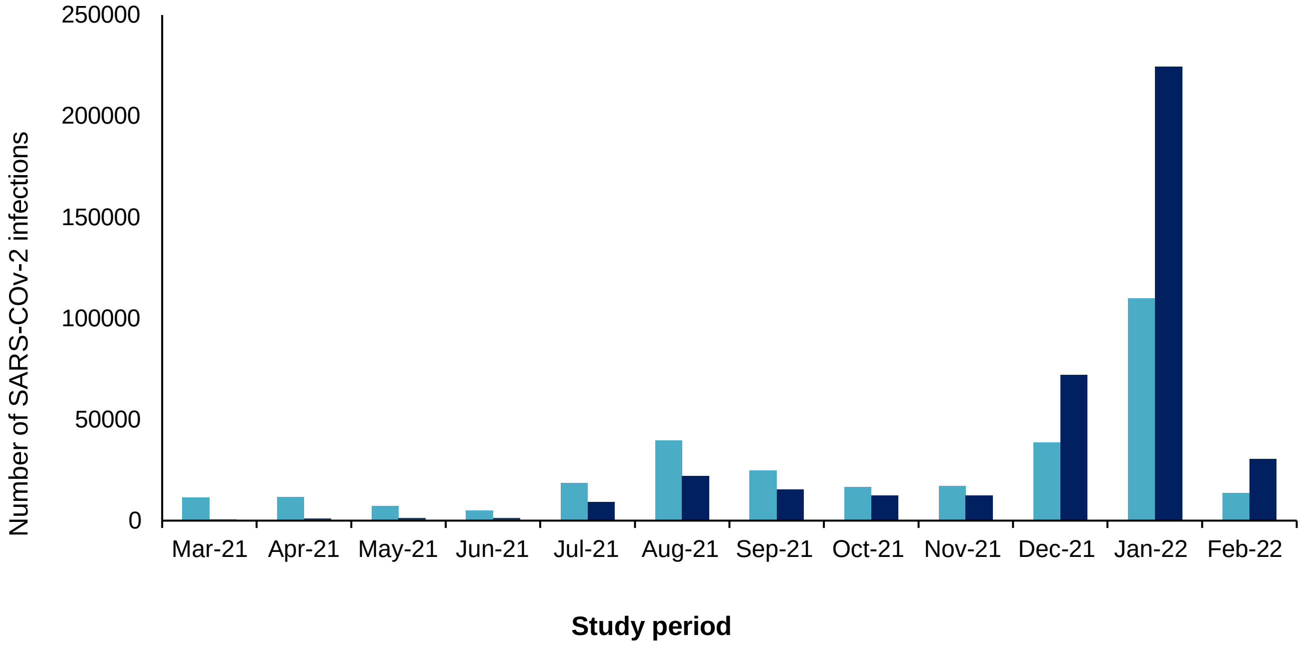

Supplementary Figure 3. Association of prior COVID-19 vaccination and risk of PCC categories 6 months following SARS-CoV-2 infection, by severity of SARS-CoV-2 infection*

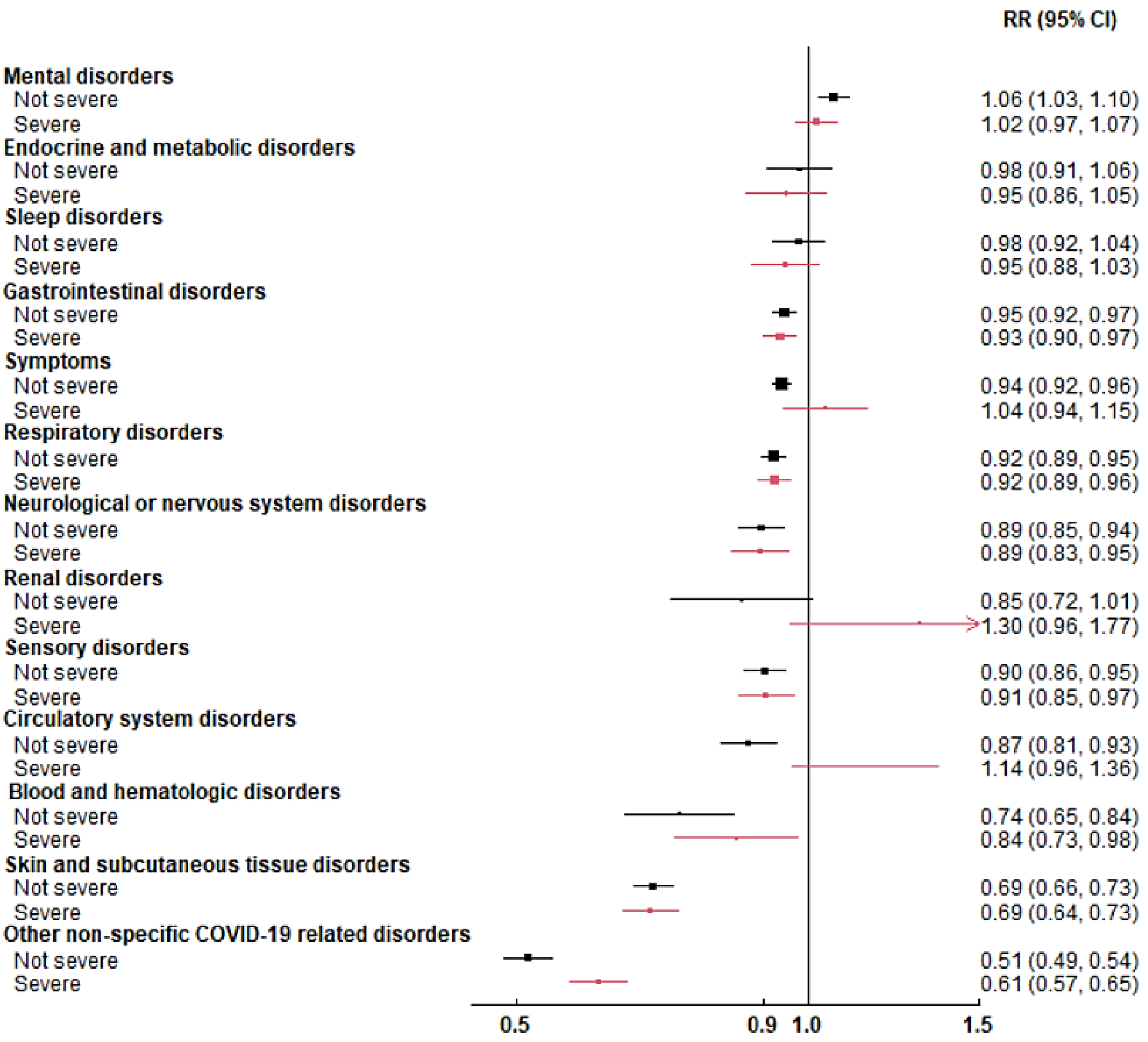

Association of prior vaccination status with Post-COVID Conditions (PCC) was estimated among 161,531 vaccinated patients matched with 161,531 unvaccinated patients on Vaccine Safety Datalink site, date of SARS-CoV-2 positive test (±7 days), age (exact year), sex, and severity of infection (hospital admission with COVID-19 diagnosis within 7 days of SARS-CoV-2 positive test). Relative risks (RR) were adjusted for matched variables and prior SARS-CoV-2 infection, race/ethnicity, Charlson comorbidity score, Medicaid status, influenza vaccination, and healthcare utilization in the year prior. Box sizes are inverse-variance weighted. PCC category 'symptoms' included headache, body ache/myalgia, fever/malaise/fatigue, lymphadenopathy, weight loss, or vertigo.

*Severity of infection was defined as hospital admission with COVID-19 diagnosis within 7 days of SARS-CoV-2 positive test

Supplementary Figure 4. Risk of PCC outcomes by time since last COVID-19 vaccine dose

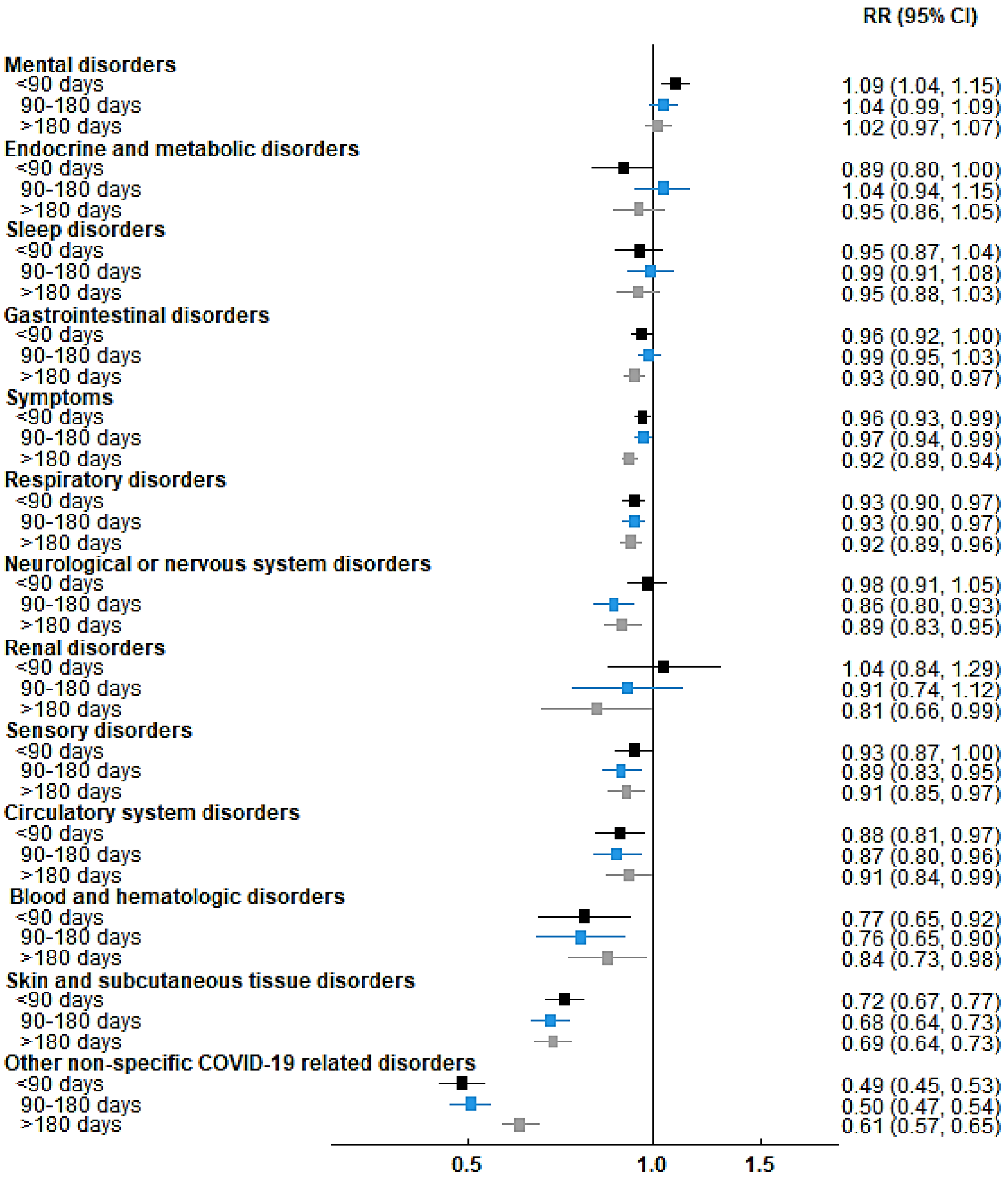

Association of prior vaccination status with Post-COVID Conditions (PCC) was estimated among 161,531vaccinated patients matched with 161,531unvaccinated patients on Vaccine Safety Datalink site, date of SARS-CoV-2 positive test (±7 days), age (exact year), sex, and severity of infection (hospital admission with COVID-19 diagnosis within 7 days of SARS-CoV-2 positive test). Relative risks (RR) were adjusted for matched variables and prior SARS-CoV-2 infection, race/ethnicity, Charlson comorbidity score, Medicaid status, influenza vaccination, and healthcare utilization in the year prior. Box sizes are inverse-variance weighted. PCC category 'symptoms' included headache, body ache/myalgia, fever/malaise/fatigue, lymphadenopathy, weight loss, or vertigo.

Supplementary Figure 5. Risk of PCC outcomes by number of COVID-19 vaccine doses received

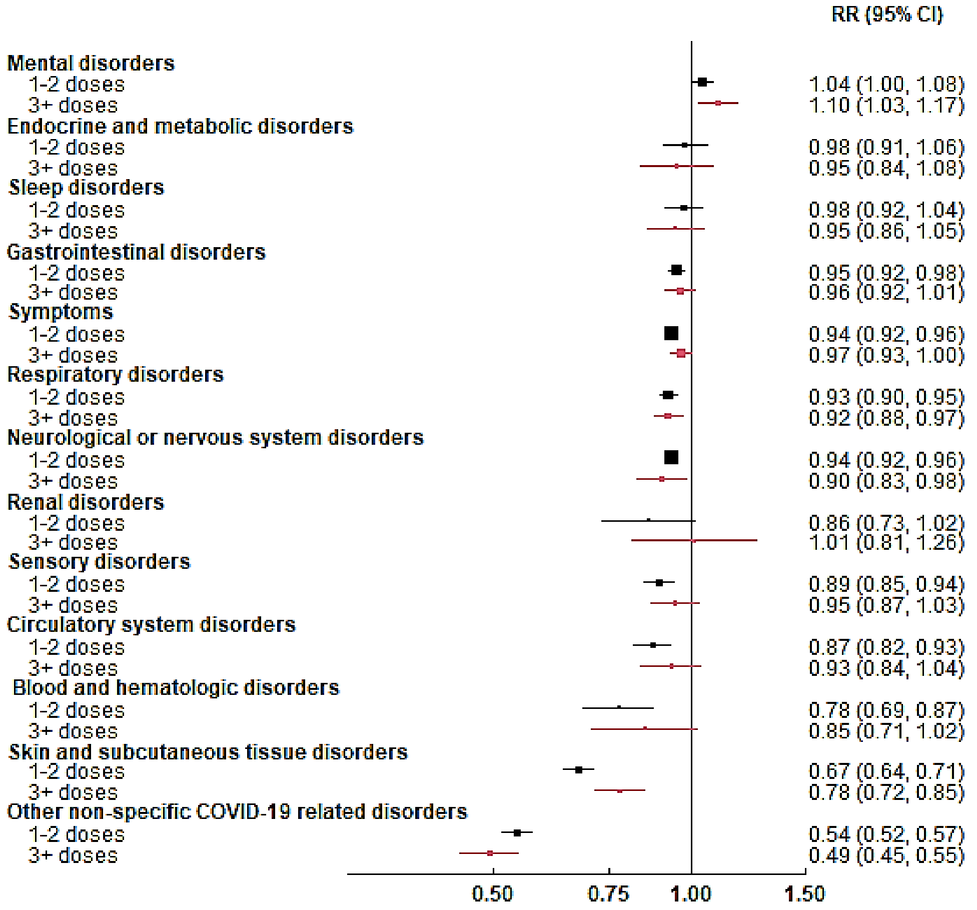

Association of prior vaccination status with Post-COVID Conditions (PCC) was estimated among 161,531vaccinated patients matched with 161,531unvaccinated patients on Vaccine Safety Datalink site, date of SARS-CoV-2 positive test (±7 days), age (exact year), sex, and severity of infection (hospital admission with COVID-19 diagnosis within 7 days of SARS-CoV-2 positive test). Relative risks (RR) were adjusted for matched variables and prior SARS-CoV-2 infection, race/ethnicity, Charlson comorbidity score, Medicaid status, influenza vaccination, and healthcare utilization in the year prior. Box sizes are inverse-variance weighted. PCC category 'symptoms' included headache, body ache/myalgia, fever/malaise/fatigue, lymphadenopathy, weight loss, or vertigo.

Supplementary Table 1. Characteristics of the unmatched study cohort, by vaccination status

	Unvaccinated (N=315022)	Vaccinated (N=402462)	Total (N=717484)	p-value	ASMD
**Age at index date, years**				<0.001	0.91
<12	99366 (31.5%)	11483 (2.9%)	110849 (15.4%)		
12-17	31000 (9.8%)	28137 (7.0%)	59137 (8.2%)		
18-24	26496 (8.4%)	34144 (8.5%)	60640 (8.5%)		
25-39	67780 (21.5%)	107387 (26.7%)	175167 (24.4%)		
40-49	36838 (11.7%)	75214 (18.7%)	112052 (15.6%)		
50-64	38291 (12.2%)	88620 (22.0%)	126911 (17.7%)		
65-74	10160 (3.2%)	36446 (9.1%)	46606 (6.5%)		
75+	5091 (1.6%)	21031 (5.2%)	26122 (3.6%)		
**Mean (SD)**	27.4 (20.6)	43.0 (18.7)	36.2 (21.0)	<0.001	0.79
**Median [Q1, Q3]**	25.0 [9.0, 42.0]	42.0 [29.0,57.0]	36.0 [19.0, 51.0]		
**Sex**				<0.001	0.12
Female	162362 (51.5%)	230679 (57.3%)	393041 (54.8%)		
Male	152660 (48.5%)	171783 (42.7%)	324443 (45.2%)		
**Race/ethnicity**				<0.001	0.25
Hispanic	113447 (36.0%)	143548 (35.7%)	256995 (35.8%)		
Asian	17864 (5.7%)	49021 (12.2%)	66885 (9.3%)		
Black	31132 (9.9%)	28258 (7.0%)	59390 (8.3%)		
White	118871 (37.7%)	142632 (35.4%)	261503 (36.4%)		
Multiple/Other/Unknown	33708 (10.7%)	39003 (9.7%)	72711 (10.1%)		
**Participating VSD Site**				<0.001	0.17
A	90242 (28.6%)	114326 (28.4%)	204568 (28.5%)		
B	13854 (4.4%)	19198 (4.8%)	33052 (4.6%)		
C	11521 (3.7%)	10365 (2.6%)	21886 (3.1%)		
D	11829 (3.8%)	7071 (1.8%)	18900 (2.6%)		
E	19151 (6.1%)	24893 (6.2%)	44044 (6.1%)		
F	5481 (1.7%)	3468 (0.9%)	8949 (1.2%)		
G	155414 (49.3%)	212913 (52.9%)	368327 (51.3%)		
H	7530 (2.4%)	10228 (2.5%)	17758 (2.5%)		
**SARS-CoV-2 variant period**				<0.001	0.70
Alpha (Mar -Jun 2021)	35419 (11.2%)	4162 (1.0%)	39581 (5.5%)		
Delta (Jul – Nov 2021)	117143 (37.2%)	71569 (17.8%)	188712 (26.3%)		
Omicron (Dec 2021 – Feb 2022)	162460 (51.6%)	326731 (81.2%)	489191 (68.2%)		
**Medicaid subsidized insurance**	63188 (20.1%)	39498 (9.8%)	102686 (14.3%)	<0.001	0.29
**Received influenza vaccine in prior 2 years**	128200 (40.7%)	271770 (67.5%)	399970 (55.7%)	<0.001	0.56
**Prior positive SARS-CoV-2 test**	24840 (7.9%)	29284 (7.3%)	54124 (7.5%)	<0.001	0.02
**Number of outpatient or virtual encounters within prior year**				<0.001	0.39
0	37962 (12.1%)	22215 (5.5%)	60177 (8.4%)		
1 - 3	108153 (34.3%)	95026 (23.6%)	203179 (28.3%)		
4 - 6	60657 (19.3%)	84751 (21.1%)	145408 (20.3%)		
7+	108250 (34.4%)	200470 (49.8%)	308720 (43.0%)		
**Weighted Charlson Comorbidity Score**				<0.001	0.31
0	265481 (84.3%)	291748 (72.5%)	557229 (77.7%)		
1 - 2	39822 (12.6%)	76693 (19.1%)	116515 (16.2%)		
3 +	9719 (3.1%)	34021 (8.5%)	43740 (6.1%)		

ASMD = Absolute standardized mean difference; SD = Standard Deviation; VSD = Vaccine Safety Datalink

Supplementary Figure 6. Risk of PCC categories 6 months following SARS-CoV-2 infection associated with prior COVID-19 vaccination among 717,484 *unmatched* SARS-CoV-2 positive tests

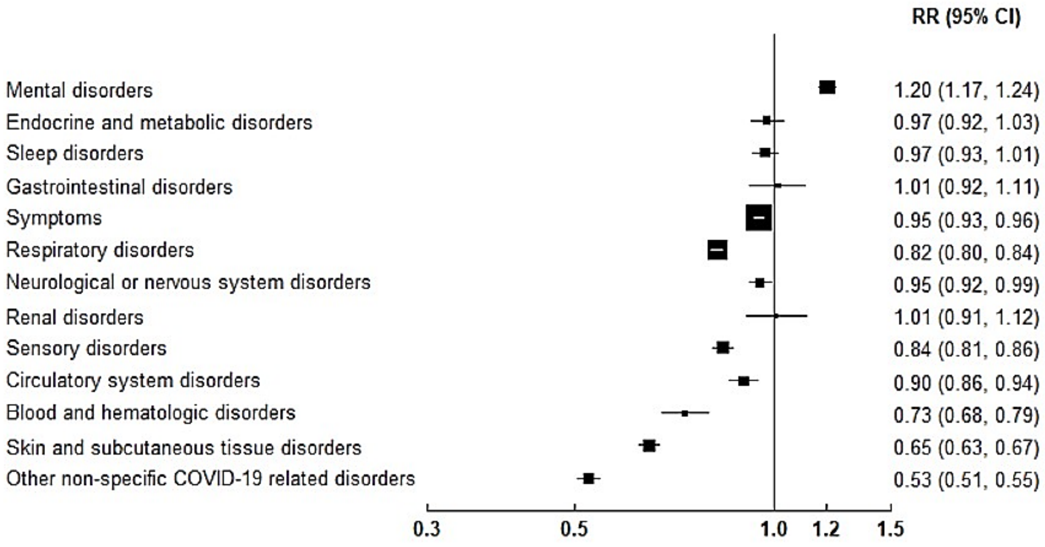

Relative risks (RR) of Post-COVID Conditions (PCC) associated with vaccination status were adjusted for Vaccine Safety Datalink site, date of SARS-CoV-2 positive test (±7 days), age (exact year), sex, and severity of infection (hospital admission with COVID-19 diagnosis within 7 days of SARS-CoV-2 positive test), prior SARS-CoV-2 infection, race/ethnicity, Charlson comorbidity score, Medicaid status, influenza vaccination, and healthcare utilization in the year prior. Box sizes are inverse-variance weighted. PCC category 'symptoms' included headache, body ache/myalgia, fever/malaise/fatigue, lymphadenopathy, weight loss, or vertigo.

Supplementary Figure 7. Risk of PCC outcomes overall and by individual sub-diagnosis category

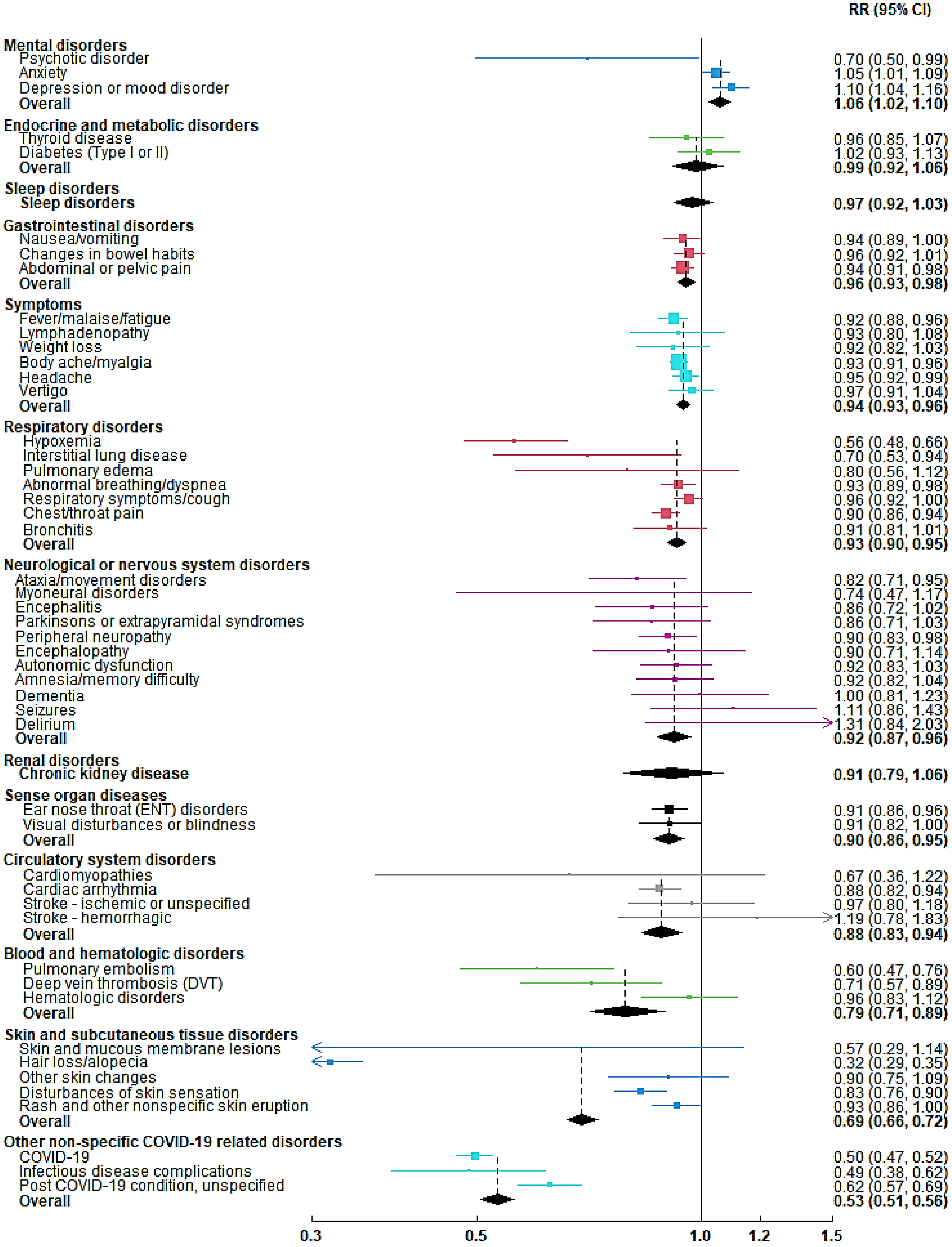

Association of prior vaccination status with Post-COVID Conditions (PCC) was estimated among 161,531 vaccinated patients matched with 161,531 unvaccinated patients on Vaccine Safety Datalink site, date of SARS-CoV-2 positive test (±7 days), age (exact year), sex, and severity of infection (hospital admission with COVID-19 diagnosis within 7 days of SARS-CoV-2 positive test). Relative risks (RR) were adjusted for matched variables and prior SARS-CoV-2 infection, race/ethnicity, Charlson comorbidity score, Medicaid status, influenza vaccination, and healthcare utilization in the year prior. Box sizes are inverse-variance weighted.

Supplementary Figure 8. Risk of PCC associated with prior COVID-19 vaccination (A) before and (B) after excluding individuals with documented PCC outcomes in the year prior to positive SARS-CoV-2 test

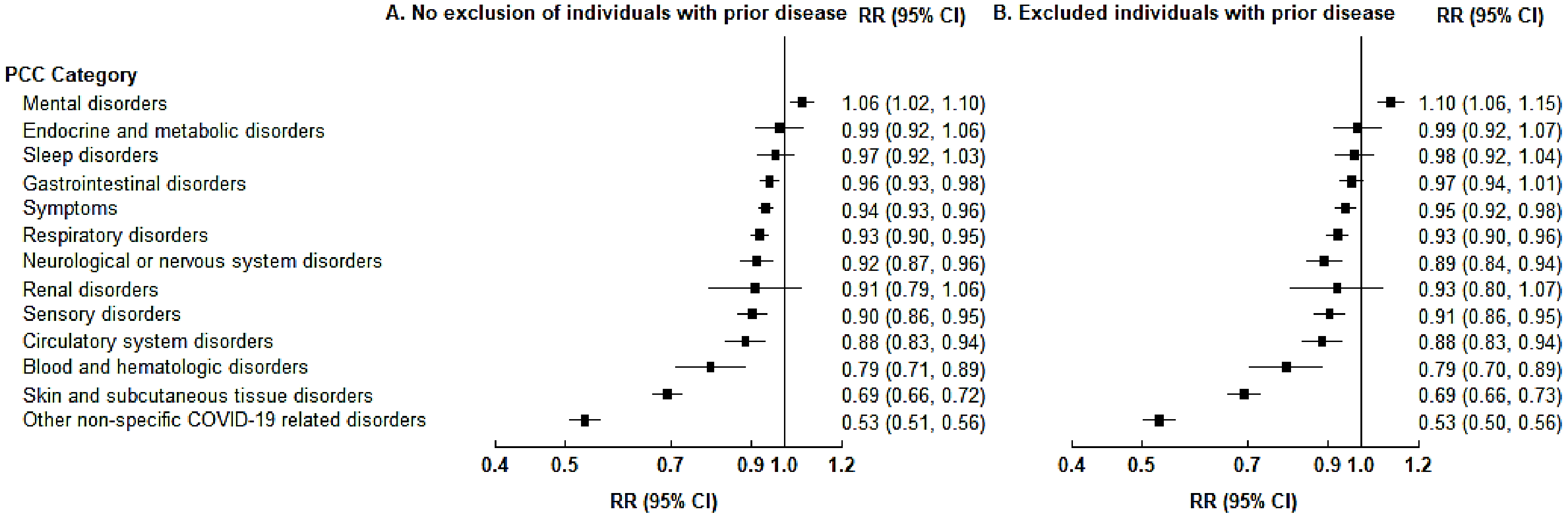

Association of prior vaccination status with Post-COVID Conditions (PCC) was estimated among 161,531 vaccinated patients with 161,531 unvaccinated patients matched on Vaccine Safety Datalink site, date of SARS-CoV-2 positive test (±7 days), age (exact year), sex, and severity of infection (hospital admission with COVID-19 diagnosis within 7 days of SARS-CoV-2 positive test). Relative risks (RR) were adjusted for matched variables and prior SARS-CoV-2 infection, race/ethnicity, Charlson comorbidity score, Medicaid status, influenza vaccination, and healthcare utilization in the year prior.

Supplementary Figure 9. Risk of PCC associated with prior COVID-19 vaccination (A) 30 days to 6 months following positive SARS-CoV-2 test and (B) 90 days – 6 months following SARS-CoV-2 test

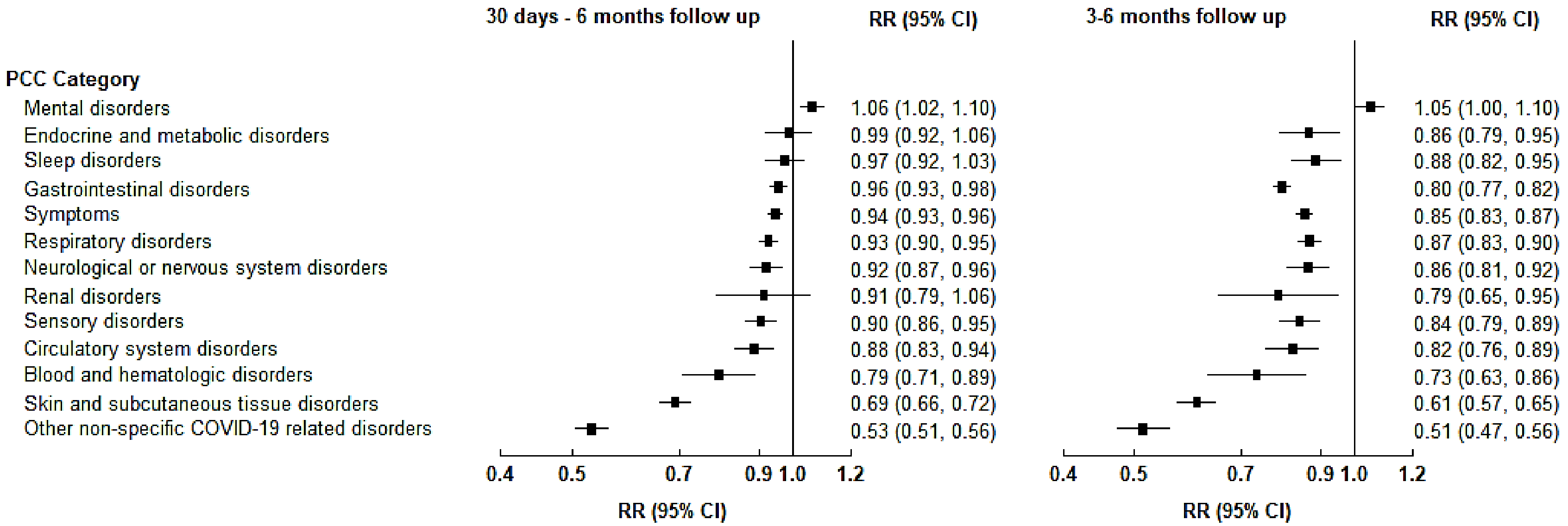

Association of prior vaccination status with Post-COVID Conditions (PCC) was estimated among 161,531 vaccinated patients with 161,531 unvaccinated patients matched on Vaccine Safety Datalink site, date of SARS-CoV-2 positive test (±7 days), age (exact year), sex, and severity of infection (hospital admission with COVID-19 diagnosis within 7 days of SARS-CoV-2 positive test). Relative risks (RR) were adjusted for matched variables and prior SARS-CoV-2 infection, race/ethnicity, Charlson comorbidity score, Medicaid status, influenza vaccination, and healthcare utilization in the year prior.

Supplementary Figure 10. Risk of Post-COVID Conditions (PCC) associated with COVID-19 vaccination status before and after matching on influenza vaccination status in the year prior to the positive SARS-CoV-2 test date: A sensitivity analysis

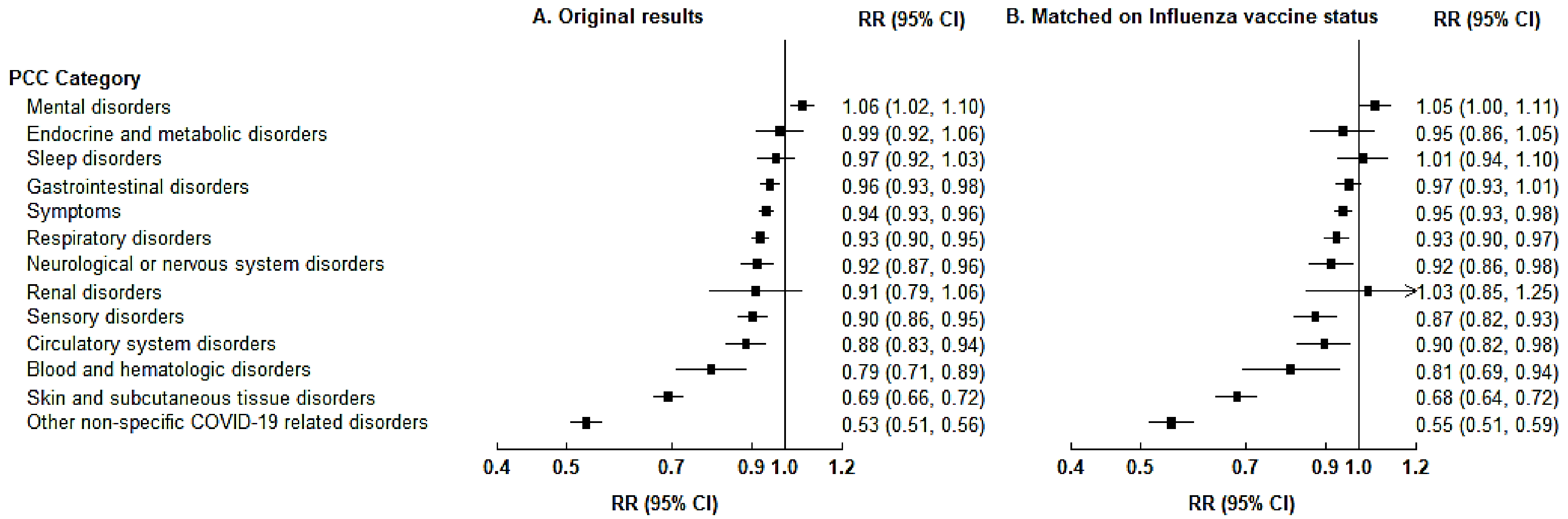

Association of prior vaccination status with Post-COVID Conditions (PCC) was estimated among vaccinated patients with unvaccinated patients matched on Influenza vaccination status in the year prior to the positive SARS-CoV-2 test date, Vaccine Safety Datalink site, date of SARS-CoV-2 positive test (±7 days), age (exact year), sex, and severity of infection (hospital admission with COVID-19 diagnosis within 7 days of SARS-CoV-2 positive test). Relative risks (RR) were adjusted for matched variables and prior SARS-CoV-2 infection, race/ethnicity, Charlson comorbidity score, Medicaid status, influenza vaccination, and healthcare utilization in the year prior.

Supplementary Figure 11. Risk of PCC associated with prior COVID-19 vaccination using (A) WHO definition and (B) NICE definition of PCC

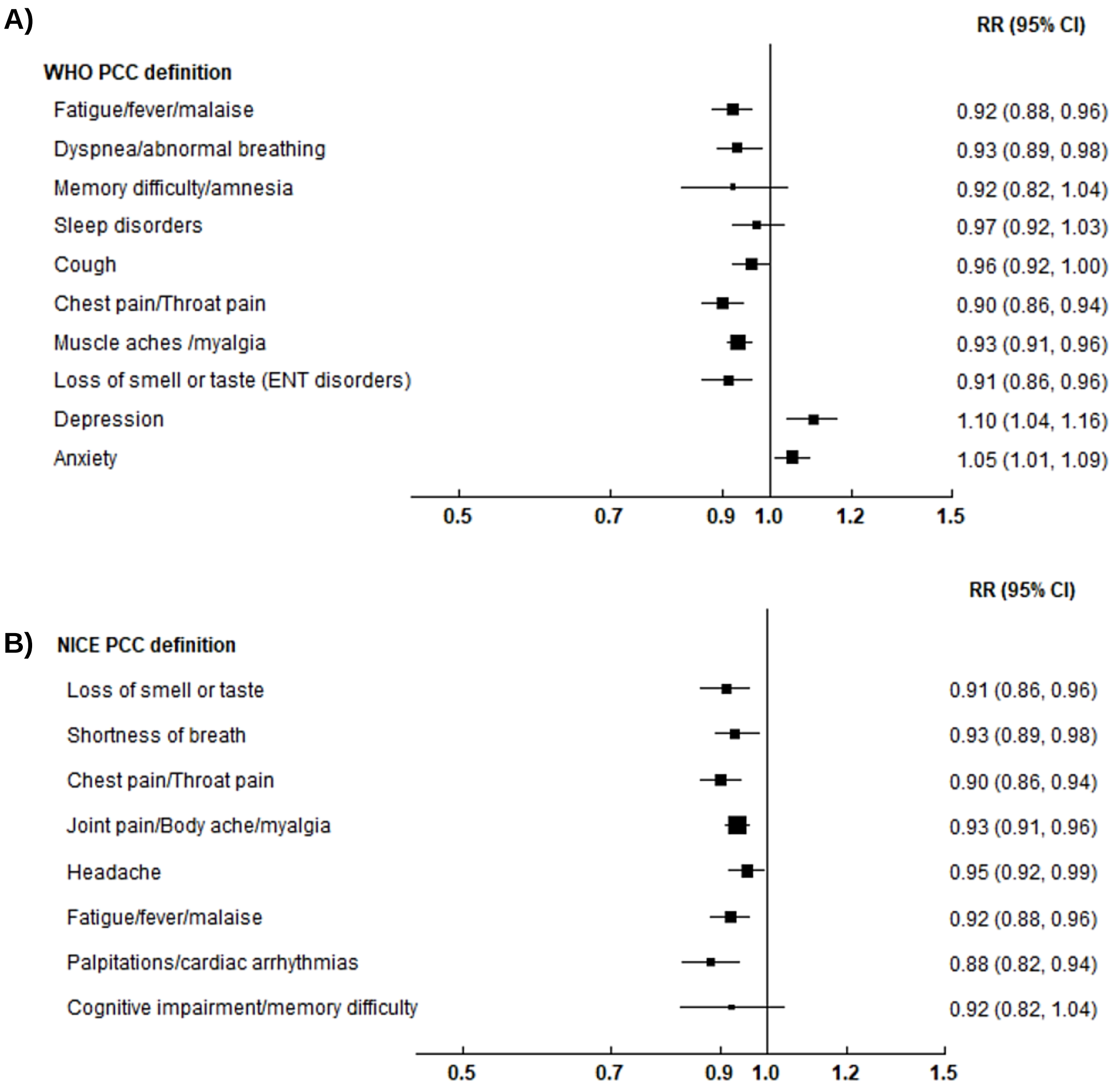

Association of prior vaccination status with Post-COVID Conditions (PCC) was estimated among 161,531 vaccinated patients with 161,531 unvaccinated patients matched on Vaccine Safety Datalink site, date of SARS-CoV-2 positive test (±7 days), age (exact year), sex, and severity of infection (hospital admission with COVID-19 diagnosis within 7 days of SARS-CoV-2 positive test). Relative risks (RR) were adjusted for matched variables and prior SARS-CoV-2 infection, race/ethnicity, Charlson comorbidity score, Medicaid status, influenza vaccination, and healthcare utilization in the year prior.

NICE = National Institute for Health and Care Excellence; WHO= World Health Organization

Supplementary Table 2. Pre-selected disease categories and ICD-10 codes used to define Post COVID Conditions

Disease category	ICD-10 code	Look-back period (prior to index date)
**Endocrine and metabolic disorders**		
Diabetes mellitus (Type I and II)	E10, E11	12 mo
Thyroid disease	E03, E06	12 mo
**Circulatory system disorders**		
Stroke – ischemic or unspecified	I63, I64, I69, G45, G46	12 mo
Stroke - hemorrhagic	I60, I61, I62	12 mo
Cardiac arrhythmias/ Postural orthostatic tachycardia syndrome (POTS)	R00, I47, I48, I49	12 mo
Cardiomyopathy, myocarditis, endocarditis	I30, I40, I51.4, B33, I51.81	12 mo
**Blood and hematologic disorders**		
Hematologic disorders	D72.81, D69	12 mo
Deep vein thrombosis (DVT)	I82	12 mo
Pulmonary embolism	I26	12 mo
**Respiratory disorders**		
Interstitial lung disease	J84	12 mo
Bronchitis	J20, J40, J41, J42	30 d
Pulmonary edema	J81	12 mo
Abnormal breathing/dyspnea	R06	30 d
Chest/throat pain	R07	30 d
Respiratory symptoms/cough	R05	30 d
Hypoxemia	R09.02	12 mo
**Mental disorders**		
Anxiety disorder	F40, F41, F42, F43, F44, F45, F48, R45	12 mo
Psychotic disorders	F20, F21, F22, F23, F24, F25, F28, F29	12 mo
Depression or mood disorders	F30, F31, F32, F33, F34, F38, F39	12 mo
**Neurological or nervous system disorders**		
Peripheral neuropathy	G50, G51, G52, G53, G54, G55, G56, G57, G58, G59, G61, G62, G64, G65	12 mo
Encephalopathy	R40.0, R44	12 mo
Seizures	G40, G41	12 mo
Dementia	F01, F02, F03, G31	12 mo
Delirium	F05	12 mo
Myoneural disorders	G72, M60	12 mo
Ataxia/movement disorders	G26, R26, R27	12 mo
Autonomic dysfunction	I95.1, G90, R55	12 mo
Parkinson’s / extrapyramidal syndromes	G21, G24, G25	12 mo
Encephalitis	A85, A86, G04, G05, R29	12 mo
Amnesia/memory difficulty	R41	12 mo

***Supplementary Table 2*.** Continued

Disease category	ICD-10 code	Look-back period (prior to index date)
**Renal disorders**		
Chronic kidney disease	N18, N19	12 mo
**Gastrointestinal disorders**		
Abdominal or pelvic pain	R10	30 d
Changes in bowel habits	K58, K59, A08, A09, R19.4, R19.7	30 d
Nausea/vomiting	R11	30 d
**Symptoms**		
Headache	G43, G44, R51	30 d
Body ache/myalgia	M02, M25, M79	30 d
Fever/malaise/fatigue	R50, R61, R53, G93.3	30 d
Lymphadenopathy	R59	12 mo
Weight loss	R63, R64	12 mo
Vertigo	A88, H81, R42	12 mo
**Skin and subcutaneous tissue disorders**		
Disturbances of skin sensation	R20	30 d
Skin and mucous membrane lesions	B09	30 d
Rash and other nonspecific skin eruption	R21	30 d
Other skin changes	R23	30 d
Hair loss/alopecia	L63, L65	12 mo
**Sensory disorders**		
Ear nose throat (ENT) disorders	H90, H91, H92, H93, J31, R43, R13	12 mo
Visual disturbances or blindness	H53, H54	12 mo
**Sleep disorders**		
Sleep disorders	G47, F51	12 mo
**Other non-specific COVID-19 related disorders**		
COVID-19	U07.1, J12.82, B97.29, B34.2	12 mo
Infectious complications	M35.81, B94	12 mo
Post COVID-19 condition, unspecified	U09.9	12 mo
