# Supplementary material for: Post-COVID conditions following COVID-19 vaccination: a retrospective matched cohort study of patients with SARS-CoV-2 infection
Source: Nat Commun. 2024 May 22;15:4101. doi: 10.1038/s41467-024-48022-9 (PMC11111703; doi:10.1038/s41467-024-48022-9)
Supplement: Supplementary file 3 — Reporting Summary [file 41467_2024_48022_MOESM3_ESM.pdf]

nature portfolio

Corresponding author(s): Debbie Malden

Last updated by author(s): 3/28/2024

Reporting Summary

Nature Portfolio wishes to improve the reproducibility of the work that we publish. This form provides structure for consistency and transparency in reporting. For further information on Nature Portfolio policies, see our Editorial Policies and the Editorial Policy Checklist.

Statistics

For all statistical analyses, confirm that the following items are present in the figure legend, table legend, main text, or Methods section.

n/a	Confirmed	
☐	☒	The exact sample size (*n*) for each experimental group/condition, given as a discrete number and unit of measurement
☒	☐	A statement on whether measurements were taken from distinct samples or whether the same sample was measured repeatedly
☐	☒	The statistical test(s) used AND whether they are one- or two-sided<br>*Only common tests should be described solely by name; describe more complex techniques in the Methods section.*
☐	☒	A description of all covariates tested
☐	☒	A description of any assumptions or corrections, such as tests of normality and adjustment for multiple comparisons
☐	☒	A full description of the statistical parameters including central tendency (e.g. means) or other basic estimates (e.g. regression coefficient) AND variation (e.g. standard deviation) or associated estimates of uncertainty (e.g. confidence intervals)
☐	☒	For null hypothesis testing, the test statistic (e.g. *F*, *t*, *r*) with confidence intervals, effect sizes, degrees of freedom and *P* value noted<br>*Give P values as exact values whenever suitable.*
☒	☐	For Bayesian analysis, information on the choice of priors and Markov chain Monte Carlo settings
☒	☐	For hierarchical and complex designs, identification of the appropriate level for tests and full reporting of outcomes
☐	☒	Estimates of effect sizes (e.g. Cohen's *d*, Pearson's *r*), indicating how they were calculated

Our web collection on statistics for biologists contains articles on many of the points above.

Software and code

Policy information about availability of computer code

Data collection	The current study was conducted within the Vaccine Safety Datalink (VSD), a research network of eight integrated healthcare systems in the United States (US) that enables standardized analysis of Electronic Health Record data. A total of 8 sites contributed data through a distributed data model (DDM) which involved running standardized SAS code script on individual-level EHR data.
Data analysis	All statistical analyses were performed using SAS statistical software version 9.4 (SAS Institute, Cary, NC) and all graphics were developed using R version 4.0.5.

For manuscripts utilizing custom algorithms or software that are central to the research but not yet described in published literature, software must be made available to editors and reviewers. We strongly encourage code deposition in a community repository (e.g. GitHub). See the Nature Portfolio guidelines for submitting code & software for further information.

Data

Policy information about availability of data

All manuscripts must include a data availability statement. This statement should provide the following information, where applicable:

- Accession codes, unique identifiers, or web links for publicly available datasets
- A description of any restrictions on data availability
- For clinical datasets or third party data, please ensure that the statement adheres to our policy

The data that support the study conclusions are unavailable for public access. Guidelines on how to access VSD data through a sharing program administered by the

National Center for Health Statistics Research Data Center (NCHSRDC) are provided here: https://www.cdc.gov/vaccinesafety/ensuringsafety/monitoring/vsd/data-sharing-guidelines.html and are subject to change.

Research involving human participants, their data, or biological material

Policy information about studies with human participants or human data. See also policy information about sex, gender (identity/presentation), and sexual orientation and race, ethnicity and racism.

Reporting on sex and gender	The current study presents sex-stratified analyses, as appropriate based on theoretical importance, such as in our baseline characteristics table. Sex was defined as male, female or other, according to Electronic Health Record documentation. Patients were excluded if they were missing information on biological sex since it is an important adjustment variable. We refer to biological sex over gender throughout the manuscript. For the main analyses, we matched on biological sex as a theoretically important variable, since males and females may differ with respect to many factors which could potentially confound the association between prior vaccination status and PCC. We feel strongly that this matching was an appropriate control measure, and additional sub-group analysis would be unnecessary. In response to an editorial suggestion, we completed a sensitivity subgroup analysis by sex and we did not find sex-specific differences across most of our associations. However, since this sex-specific analyses was not decided a-priori, we have not included this result in our manuscript. Indeed the journal discourages post-hoc sex- and gender-based analysis, and hence we are aligned with this recommendation.
Reporting on race, ethnicity, or other socially relevant groupings	Race and/or ethnicity was defined in five mutually exclusive categories: Hispanic, Black, Asian, White, and Other/Unknown, as documented in participants individual-level electronic health records. Where appropriate, race/ethnicity-stratified analyses was presented in the results, and all multi-variate analysis adjusts for race/ethnicity.
Population characteristics	Among all COVID-19 cases included in the analysis, the mean age was 37.1 (standard deviation [SD] 17.9) years, 54.5% were female. Most of the study population identified as either Hispanic (36.2%) or White (37.1%) race/ethnicity. Individuals of Black and Asian race/ethnicity each accounted for less than 10% of the study population. Compared with unvaccinated patients, vaccinated patients were more likely to be Asian (12.1% vs. 4.0%) and less likely to have Medicaid subsidized insurance (11.0% vs. 16.3%;).
Recruitment	The study population included patients of all ages enrolled at a VSD site with at least one documented SARS-CoV-2 positive test result (PCR or antigen) from March 1st, 2021, to February 28th, 2022, and 1-year continuous health plan membership (allowing for a 31-day administrative gap) prior to their SARS-CoV-2 positive test date, with a waiver for informed consent. There is the potential for selection bias based on the need for full continuous healthcare insurance coverage and a positive SARS-CoV-2 test, which may limit the representativeness of the study population. Also, patients must have survived for at least 30 days following the positive SARS-CoV-2 test. However, we match on severity of acute illness and time of test, which should limit the impact of these biases on the overall study findings.
Ethics oversight	This study was reviewed and approved by institutional review boards of all participating health care organization sites (8 healthcare systems) with a waiver of informed consent and was conducted consistent with federal law and CDC policy. See, for example 45 C.F.R. part 46.101(c); 21 C.F.R. part 56.

Note that full information on the approval of the study protocol must also be provided in the manuscript.

Field-specific reporting

Please select the one below that is the best fit for your research. If you are not sure, read the appropriate sections before making your selection.

☒ Life sciences ☐ Behavioural & social sciences ☐ Ecological, evolutionary & environmental sciences

For a reference copy of the document with all sections, see nature.com/documents/nr-reporting-summary-flat.pdf

Life sciences study design

All studies must disclose on these points even when the disclosure is negative.

Sample size	323,062. Sample size was determined based on the number of positive SARS-CoV-2 tests meeting the study inclusion and exclusion criteria over the study period at the 8 VSD sites, respectively. We did not estimate a required sample size for this study given the large number of SARS-CoV-2 infections occurring over the study period at each of the VSD regions. We did not anticipate facing issues with statistical power given the large nature of the integrated healthcare systems included in this study.
Data exclusions	To ensure sufficient time for immunological response following vaccination and to accurately distinguish between PCC and post-vaccine reactions, patients were excluded if they received a COVID-19 vaccine within 14 days prior to or within 30 days after their SARS-CoV-2 positive test date. Patients were also excluded if they were missing information on age or sex since these were important adjustment variables. In addition, if patients received either a Janssen (Johnson & Johnson) COVID-19 vaccine or a COVID-19 vaccination not routinely administered in the US, they were not included in the analysis since it was thought that these patients would systematically differ from mRNA vaccine recipients with respect to key clinical and demographic characteristics.
Replication	To assess the robustness of estimates among the total population of all SARS-CoV-2 positive patients over the study period, we conducted an unmatched sensitivity analysis with the same covariates included in the multi-variable models as those listed above. To assess directional concordance of effect estimates for sub-conditions with the overall effect size estimates for PCC categories, we repeated the main analysis for all 51 sub-condition. To limit the impact of type I error, Bonferroni correction was applied to the main analysis, with 2-sided p-values at a level of significance of 0.004 (calculated as 0.05/13). All sensitivity analyses were successful in demonstrating the robustness of the study results.

Randomization	This study was a retrospective matched cohort study using electronic health records from patients of all ages with SARS-CoV-2 positive tests (PCR or antigen) during March 2021-February 2022. Vaccinated and unvaccinated COVID-19 cases were matched on location, test date, severity of acute infection, age, and sex.
Blinding	Blinding was not relevant for this study since it was a retrospective cohort study using existing clinical data.

Reporting for specific materials, systems and methods

We require information from authors about some types of materials, experimental systems and methods used in many studies. Here, indicate whether each material, system or method listed is relevant to your study. If you are not sure if a list item applies to your research, read the appropriate section before selecting a response.

Materials & experimental systems

n/a	Involved in the study	
☒	☐	Antibodies
☒	☐	Eukaryotic cell lines
☒	☐	Palaeontology and archaeology
☒	☐	Animals and other organisms
☐	☒	Clinical data
☒	☐	Dual use research of concern
☒	☐	Plants

Methods

n/a	Involved in the study	
☒	☐	ChIP-seq
☒	☐	Flow cytometry
☒	☐	MRI-based neuroimaging

Clinical data

Policy information about clinical studies

All manuscripts should comply with the ICMJE guidelines for publication of clinical research and a completed CONSORT checklist must be included with all submissions.

Clinical trial registration	N/A
Study protocol	The study protocol is available on the VSD study website: https://www.cdc.gov/vaccinesafety/pdf/SCK1344-Long-COVID-VSD-Proposal-508.pdf
Data collection	This study was a retrospective matched cohort study using electronic health records from patients of all ages with SARS-CoV-2 positive tests (PCR or antigen) during March 2021-February 2022. All individuals in the study were followed from 30 days to 6 months after the date of positive SARS-CoV-2 test (i.e., index date).
Outcomes	The outcome was defined as any documentation of new-onset pre-specified PCC outcomes occurring ≥30 days after the SARS-CoV-2 positive test (i.e., following the acute stage of infection). To determine new-onset PCC status, pre-existing PCC conditions were identified during outcome-specific look-back periods. If a pre-existing PCC condition was identified within this look-back period, re-occurrence within the follow-up period did not contribute to the analyses. For example, if an individual in the study had diabetes mellitus documented in their EHR within twelve months prior to the index date, diabetes mellitus codes identified during the study follow-up period were not identified as a new-onset PCC outcome in the analysis. This approach was favored over the exclusion of individuals with pre-existing PCC events prior to the study period because it ensured that the study population was consistent across all PCC events studied.

Plants

Seed stocks	N/A
Novel plant genotypes	N/A
Authentication	N/A
